# Long noncoding RNA LINC00673 epigenetically suppresses KLF4 by interacting with EZH2 and DNMT1 in gastric cancer

**DOI:** 10.18632/oncotarget.20980

**Published:** 2017-09-18

**Authors:** Ming-Chen Ba, Hui Long, Shu-Zhong Cui, Yuan-Feng Gong, Zhao-Fei Yan, Yin-Bing Wu, Yi-Nuo Tu

**Affiliations:** ^1^ Intracelom Hyperthermic Perfusion Therapy Center, Affiliated Cancer Hospital and Institute of Guangzhou Medical University, Guangzhou 510095, P.R. China; ^2^ Department of Pharmacy, Guangzhou Dermatology Institute, Guangzhou 510095, P.R. China

**Keywords:** long noncoding RNA, LINC00673, DNMT1, EZH2, KLF4

## Abstract

Long non-coding RNAs (lncRNAs), a variety of transcripts without protein coding ability, have recently been reported to play vital roles in gastric cancer (GC) development and progression. However, the biological role of long non-coding RNA LINC00673 in GC is not fully known. In the study, we found that LINC00673 expression was dramatically higher in gastric cancer tissues compared with adjacent normal tissues, and positively associated with lymph node metastasis, distant metastasis and TNM stage in patients. Higher LINC00673 expression predicted poor disease-free survival (DFS) and overall survival (OS) in GC patients. By univariate and multivariate Cox analysis, the results confirmed that higher LINC00673 expression was an independent risk factor of prognosis in patients. Knockdown of endogenous LINC00673 significantly inhibited cell proliferation, colony formation number, cell migration and invasion in GC. Furthermore, knockdown of endogenous LINC00673 reduced the expression levels of PCNA, CyclinD1 and CDK2 in GC cells. RNA immunoprecipitation (RIP) and chromatin immunoprecipitation (ChIP) proved that LINC00673 suppressed KLF4 expression by interacting with EZH2 and DNMT1 in GC cells. Moreover, we confirmed that LINC00673 promoted cell proliferation and invasion by partly repressing KLF4 expression in GC. Taken together, these results indicated that LINC00673 may be a prognostic biomarker and therapeutic target for GC patients.

## INTRODUCTION

Gastric cancer (GC) is one of the most common types of cancer with high morbidity and mortality worldwide [1]. In China, GC is the second leading cause of cancer related death, approximately 679000 new cases of GC and 498000 GC associated deaths in 2015 [2]. Despite larger advances in diagnosis and treatment of GC, the overall survival rate for advanced GC patients remain still poor with a very low 5-year overall survival (OS) rate (<10%), due to the limited therapeutic options, tumor metastasis and recurrence [3]. Thus, it is essential to identify new biological makers for predicting prognosis and therapeutic targets for GC.

Recent literatures have reported that long non-coding RNAs (lncRNAs) dysregulated in a variety of tumors and function as critical regulators of cancer progression. For example, downregulated expression of the long non-coding RNA LINC00261 predicts poor prognosis for gastric cancer patients and suppresses tumor metastasis by regulating the epithelial-mesenchymal transition (EMT) process [4]. Long non-coding RNA HULC served as a novel serum biomarker for diagnosis and prognostic prediction in gastric cancer [5]. Over-expression of long non-coding RNA HOTTIP promotes tumor invasion and predicts a poor prognosis in gastric cancer patients [6]. Over-expression of long non-coding antisense RNA KRT7-AS promotes gastric cancer progression via increasing KRT7 expression level [7]. These studies indicated that lncRNAs are responsible for the progression of GC.

Long non-coding RNA00673 was found to be involved in tumor progression. Up-regulation of long intergenic non-coding RNA 00673 promotes tumor proliferation via LSD1 interaction and repression of NCALD in non-small-cell lung cancer [8]. Over-expression long non-coding RNA LINC00673 is associated with poor prognosis and promotes invasion and metastasis in tongue squamous cell carcinoma [9]. In gastric cancer progression, Long non-coding RNA LINC00673 is activated by SP1 and exerts oncogenic properties by interacting with LSD1 and EZH2 [10]. However, the biological function of LINC00673 in GC is not fully investigated.

In the study, we confirmed that LINC00673 was dramatically higher in gastric cancer tissues compared to adjacent normal tissues and positively associated with poor survival outcome in patients. Knockdown of LINC00673 significantly suppressed cell proliferation, migration and invasion in GC. Besides, we confirmed that LINC00673 promoted cell proliferation and invasion by inhibiting KLF4 expression via interacting with EZH2 and DNMT1 in GC. Thus, our results indicated that LINC00673 may be a prognostic biomarker and therapeutic target for GC patients.

## RESULTS

### LINC00673 expression is significantly upregulated in GC tissues

**Figure 1:**
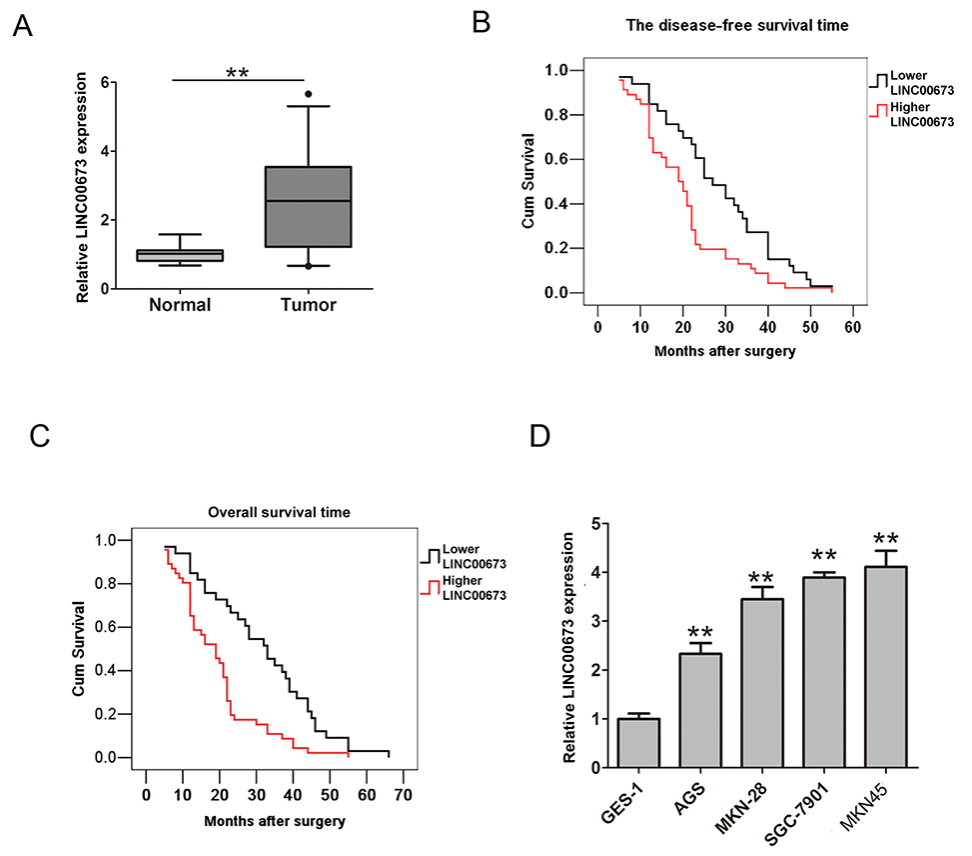
LINC00673 was significantly higher expression in GC tissues and cells **(A)** The relative expression of LINC00673 in GC tissues and adjacent normal tissues was analyzed by qRT-PCR analysis (n=79). **(B)** Representative relationship between the relative expression of LINC00673 and DFS in patients. **(C)** Representative relationship between the relative expression of LINC00673 and OS in patients. **(D)** The relative expression of LINC00673 in four GC cells and GES-1 cells were examined by qRT-PCR. The mean values and SD were evaluated from triplicates of a representative experiment, ^**^p < 0.05.

Firstly, we assessed the mRNA expression levels of LINC00673 in 79 cases of human GC tissues and adjacent normal tissues by qRT-PCR analysis. As shown in Figure 1A, compared with adjacent normal tissues, the relative mRNA expression levels of LINC00673 were significantly higher in GC tissues. Clinicpathological characteristics were shown in Table 1. According to the median expression of LINC00673, patients were classified into two groups: higher LINC00673 expression group and lower LINC00673 expression group. Furthermore, we examined the correlation between LINC00673 mRNA expression and clinicpathological characteristics in patients. The statistical analysis results showed that higher LINC00673 expression in patients was positively correlated with lymph node metastasis, distance metastasis and advanced TNM stage (P<0.05, Table 1). However, no statistical significance was found between LINC00673 expression and other parameters such as age, sex, tumor size, and so on (P>0.05, Table 1). Besides, we investigated the relationship between LINC00673 expression and disease-free survival (DFS) and overall survival (OS) in GC patients. The survival curve by the Kaplan-Meier analysis and log-rank test confirmed that higher LINC00673 expression levels were negatively association with disease-free survival (DFS) (log rank=7.558, P<0.05, Figure 1B) and overall survival (OS) (log rank=13.008, P<0.05, Figure 1C) in GC patients. Additionally, univariate and multivariate Cox model demonstrated that lymph node metastasis, distance metastasis, advanced TNM stage and higher LINC00673 expression were identified as independent risk factors for DFS (Table 2) or OS (Table 3) in patients. These results implied that LINC00673 overexpression may be a potential biological marker for predicting prognostic in GC patients.

**Table 1:** The association between expression of LINC00673 and clinical characteristics in 79 gastric cancer specimens

Characteristics	Patients (n=79)	LINC00673 expression		p-value
		Lower (n=33)	Higher (n=46)	
Sex				0.154
Male	48	17	31	
Female	31	16	15	
Age (years)				0.246
≤55	49	18	31	
>55	30	15	15	
Depth of tumor				0.096
T1 and T2	44	22	22	
T3 and T4	35	11	24	
Tumor size				0.234
<5cm	49	23	26	
>5cm	30	10	20	
Histological differentiation				0.315
High, middle	55	25	30	
Low	24	8	16	
Lymph node metastasis				0.001^a^
Negative	29	20	9	
Positive	50	13	37	
Distance metastasis				0.017^a^
Negative	45	24	21	
Positive	34	9	25	
TNM stage				0.001^a^
I and II	29	19	10	
III and IV	50	14	36	

^a^ p-value <0.05 was considered statistically significant(Chi-square test between groups).

**Table 2:** Univariate and multivariate Cox proportional hazard analysis for DFS

Variables	Univariate analysis			Multivariate analysis		
	HR	95%CI	p-value	HR	95%CI	p-value
Sex	1.212	0.808-1.665	0.228			
Age	1.030	0.466-1.157	0.556			
Depth of tumor	1.134	0.480-1.788	0.322			
Tumor size	0.909	0.511-1.660	0.665			
Histological differentiation	1.121	0.408-1.776	0.504			
Lymph node metastasis	2.667	1.454-4.388	0.001^a^	2.274	1.220-3.664	0.001^a^
Distance metastasis	2.532	1.556 -4.202	0.001^a^	2.321	1.247-3.797	0.001^a^
TNM stage	2.738	1.023 -4.699	0.001^a^	2.335	1.306-4.079	0.001^a^
LINC00673	3.156	1.448-5.228	0.001^a^	2.943	1.226-4.208	0.001^a^

^a^p-value <0.05 was considered statistically significant, CI: confidence intervals.

**Table 3:** Univariate and multivariate Cox proportional hazard analysis for OS

Variables	Univariate analysis			Multivariate analysis		
	HR	95%CI	p-value	HR	95% CI	p-value
Sex	1.008	0.611-1.554	0.355			
Age	0.834	0.341-1.699	0.619			
Depth of tumor	1.011	0.513-1.868	0.441			
Tumor size	1.115	0.533-1.989	0.306			
Histological differentiation	0.942	0.488-1.669	0.558			
Lymph node metastasis	2.566	1.133-4.116	0.001^a^	2.301	1.099-3.903	0.001^a^
Distance metastasis	2.707	1.522-4.233	0.001^a^	2.445	1.255-3.991	0.001^a^
TNM stage	2.442	1.232-4.056	0.001^a^	2.122	1.220-3.760	0.001^a^
LINC00673	2.989	1.126-5.178	0.001^a^	2.556	1.007-4.543	0.001^a^

^a^p-value <0.05 was considered statistically significant, CI: confidence intervals.

### Knockdown of LINC00673 suppresses cell proliferation in GC cells

**Figure 2:**
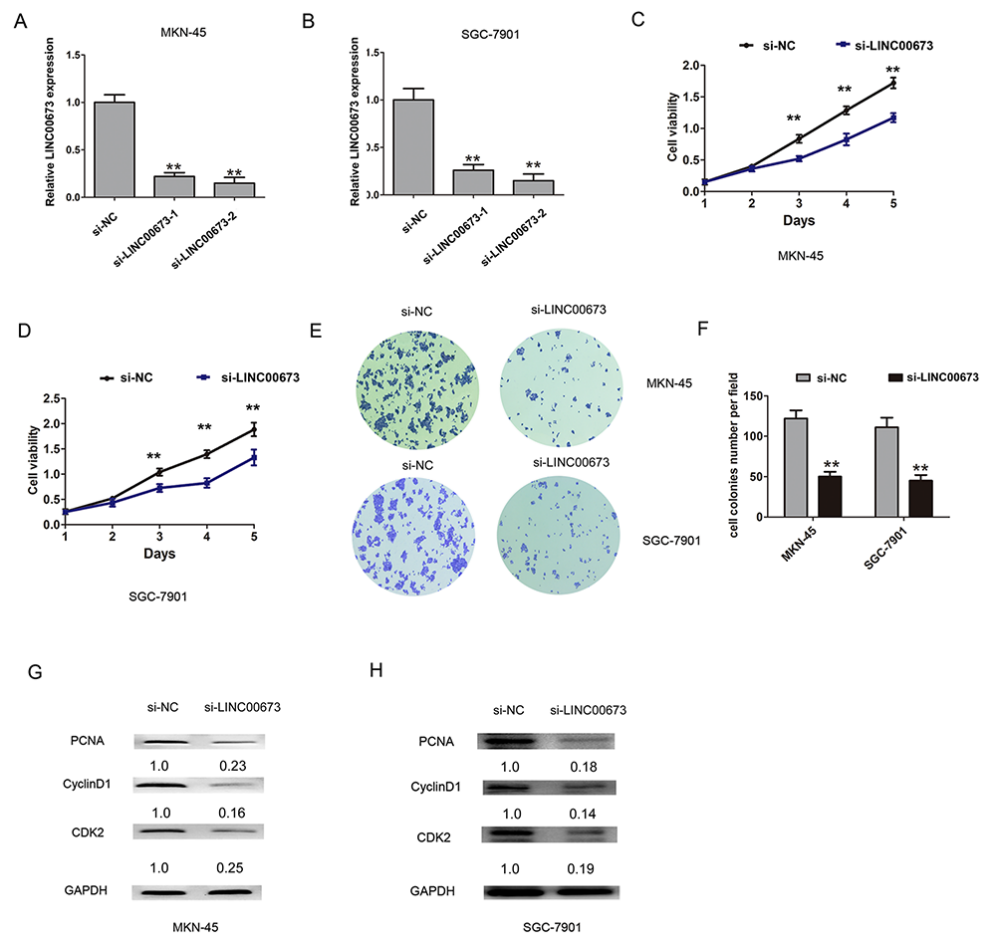
Knockdown of LINC00673 suppressed cell proliferation in GC cells **(A-B)** The relative expression of LINC00673 was assessed by qRT-PCR after LINC00673 knockdown in MKN-45 and SGC-7901 cells compared with the si-NC group. **(C-D)** Representative CCK8 analysis of cell viability after LINC00673 knockdown in MKN-45 and SGC-7901 cells. **(E-F)** Cell colony formation assay was performed and colonies number was calculated after LINC00673 knockdown in MKN-45 and SGC-7901 cells. **(G-H)** The relative protein expression of PCNA, CyclinD1 and CDK2 were detected by western blot after LINC00673 knockdown in MKN-45 and SGC-7901 cells. The mean values and SD were evaluated from triplicates of a representative experiment, ^**^p < 0.05.

To examine the LINC00673 whether functionally involved in GC progression, we measured the expression of LINC00673 in GC cell lines (MKN45, AGS, MKN-28 and SGC-7901) and an immortalized normal gastric epithelial cell line (GES-1). The results showed that LINC00673 expression were significantly elevated in GC cells, compared with GES-1 cells (Figure 1D). We used two chemically synthesized siRNAs targeting LINC00673 to knock down endogenous LINC00673 in two higher LINC00673 expression cell lines (MKN45 and SGC-7901 cells). The results showed that si-LINC00673-2 had higher efficiency of interference than si-LINC00673-1 in MKN-45 and SGC-7901 cells (Figure 2A-2B). Based on the above results, we transfected with si-LINC00673-2 into GC cells for the following experiments. CCK-8 and colony formation assays were performed to assess the effects of LINC00673 on cell proliferation in GC. The results from CCK8 assay confirmed that LINC00673 knockdown significantly inhibited cell viability compared with the control group in MKN-45 and SGC-7901 cells (Figure 2C-2D). Cell colony formation assay revealed that cell colonies grew significantly slower in LINC00673 knockdown group, compared with the si-NC group in MKN-45 and SGC-7901 cells (Figure 2E-2F). Accordingly, cell proliferation regulated protein expression levels of PCNA, Cyclin D1 and CDK2 were downregulated after knockdown of LINC00673 in MKN-45 and SGC-7901 cells, compared with the si-NC group (Figure 2G-2H). These findings indicated that LINC00673 silencing dramatically suppressed GC cell proliferation.

### Knockdown of LINC00673 suppresses cell migration and invasion in GC

**Figure 3:**
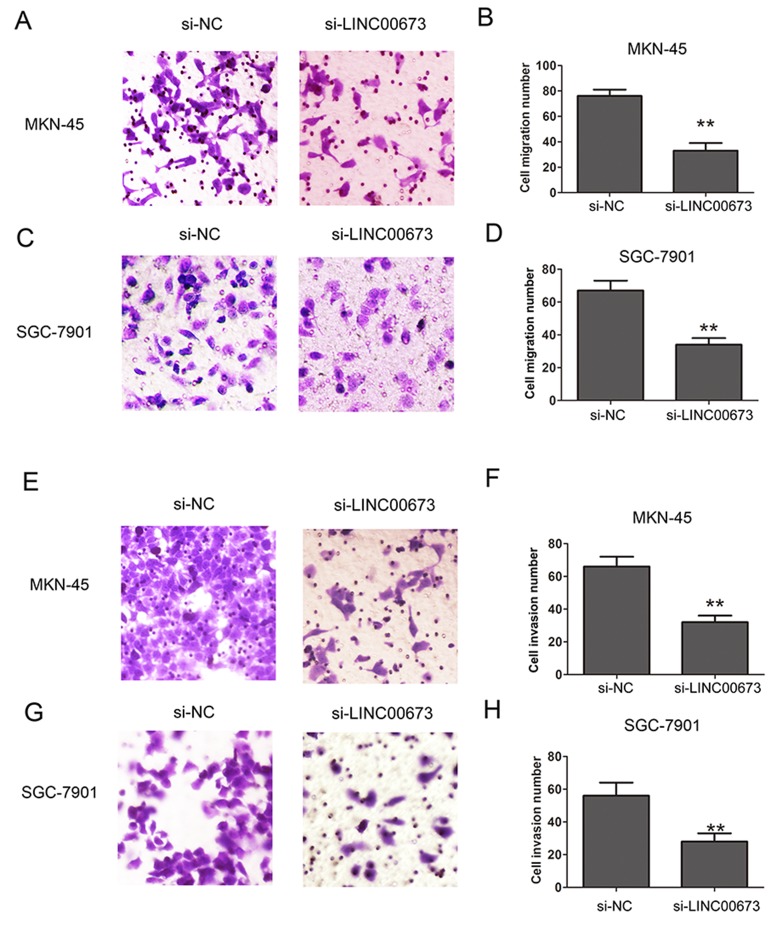
Knockdown of LINC00673 suppressed cell migration and invasion in GC **(A-D)** Cell migration ability was assessed by transwell assay after LINC00673 knockdown in MKN-45 and SGC-7901 cells. **(E-H)** Cell invasion ability was assessed by transwell assay after LINC00673 knockdown in MKN-45 and SGC-7901 cells. The mean values and SD were evaluated from triplicates of a representative experiment, ^**^p < 0.05.

To determine whether LINC00673 expression affected cell migration and invasion in GC, transwell migration and invasion assays were performed. The results confirmed that LINC00673 knockdown significantly reduced cell migration number, compared with the si-NC group in MKN-45 and SGC-7901 cells by transwell cell migration assay (Figure 3A-3D). Similarly, transwell cell invasion assay demonstrated that LINC00673 knockdown significantly reduced cell invasive number, compared with si-NC group in MKN-45 and SGC-7901 cells (Figure 3E-3H). These results suggested that LINC00673 silencing suppressed GC cell migration and invasion ability.

### LINC00673 suppresses KLF4 expression via interacting with EZH2 and DNMT1 in GC cells

**Figure 4:**
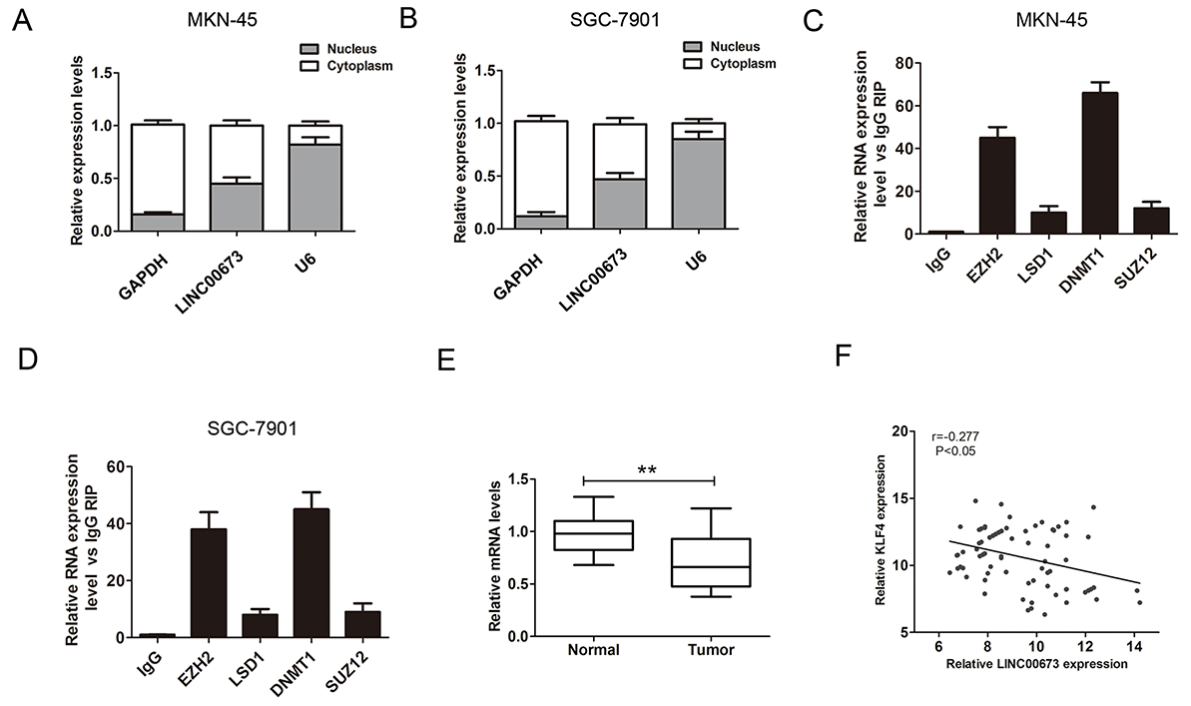
LINC00673 bound to EZH2 and DNMT1 in GC cells **(A-B)** QRT-PCR were used to detect the percentage of LINC00673 expression, U1, and GAPDH in the cytoplasm and nuclear fractions of MKN-45 and SGC-7901 cells. **(C-D)** RNA immunoprecipitates were performed using antibodies with IgG, EZH2, LSD1, DNMT1 and SUZ12 by qRT-PCR. LINC00673 RNA expression levels are presented as fold enrichment values relative to IgG immunoprecipitates. **(E)** The relative expression levels of KLF4 was detected in GC tissues and adjacent normal tissues by QRT-PCR (n=79). **(F)** Association between KLF4 expression and LINC00673 expression in GC tissues by Spearman correlation coefficient analysis. The mean values and SD were evaluated from triplicates of a representative experiment, ^**^p < 0.05.

Studies have revealed that lncRNAs could regulate key downstream mediators through RNA binding proteins including EZH2, PRC2, STAU1, DNMT1 and LSD1 [11, 12]. To confirm the speculation, we firstly detected the distribution of LINC00673 in both cytoplasm and nucleus in GC cells. The result demonstrated that LINC00673 located at cytoplasm and nucleus in MKN-45 and SGC-7901 cells (Figure 4A-4B). Furthermore, we performed RIP assay using antibodies with IgG, EZH2, DNMT1, LSD1 and SUZ12 in MKN-45 and SGC-7901 cells. The results indicated that LINC00673 could directly bind to EZH2 and DNMT1 in MKN-45 and SGC-7901 cells (Figure 4C-4D).

**Figure 5:**
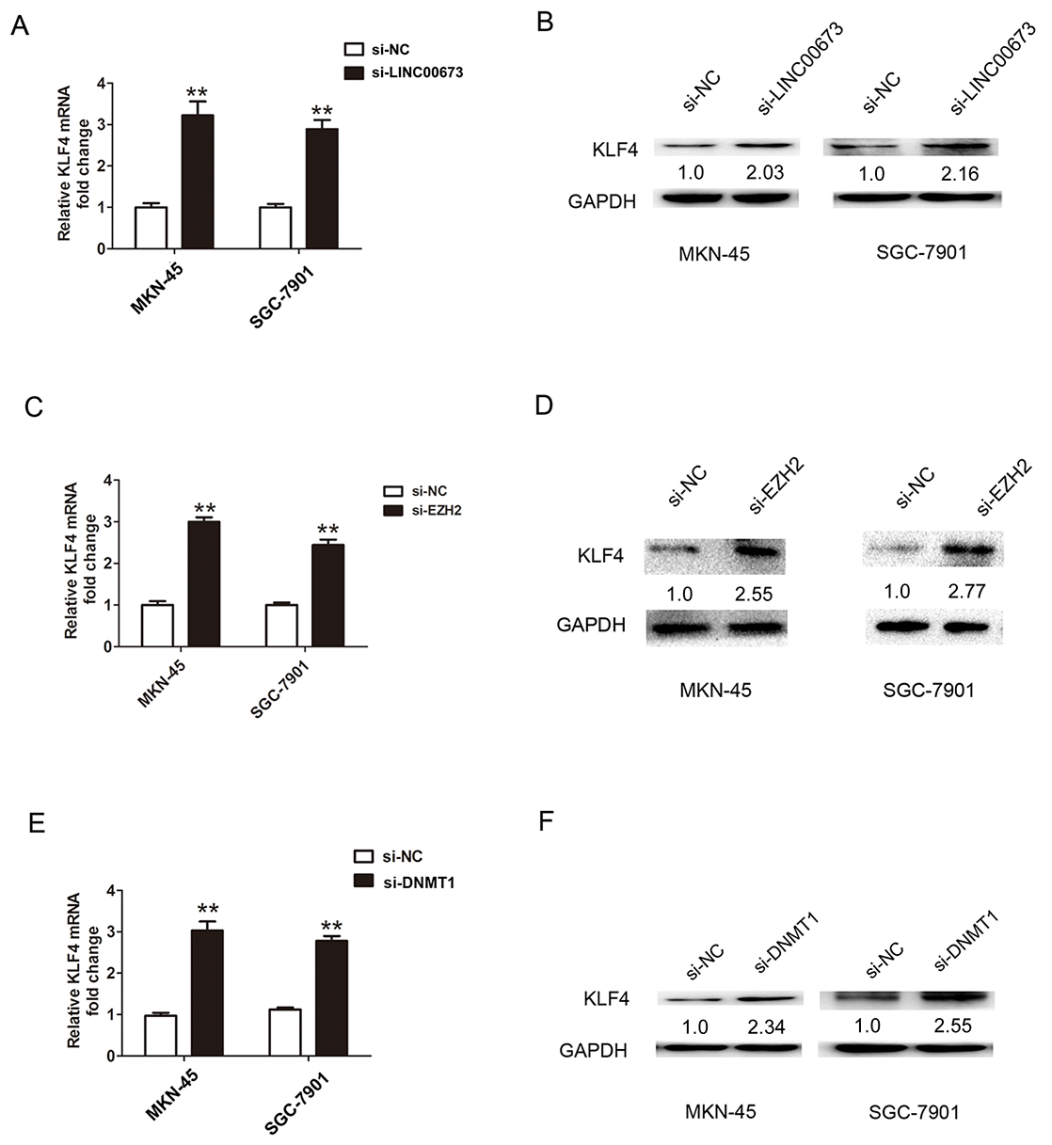
LINC00673 suppressed KLF4 expression via interacting with EZH2 and DNMT1 in GC cells **(A-B)** The relative transcription and protein levels of KLF4 after LINC00673 silencing in MKN-45 and SGC-7901 cells. **(C-F)** The relative transcription and protein levels of KLF4 after EZH2 or DNMT1 silencing in MKN-45 and SGC-7901 cells. The mean values and SD were evaluated from triplicates of a representative experiment, ^**^p < 0.05.

KLF4 play a key tumor suppressor role including regulating cell proliferation, migration and invasion in GC [13]. DNMT1 could represses Krüppel-like factor 4(KLF4) through bind to its promoter regions and contributes to EMT in renal fibrosis [14]. QRT-PCR results showed that KLF4 expression was lower in GC tissues compared with adjacent normal tissues (Figure 4E). Spearman correlation coefficient showed that LINC00673 was reversely associated with KLF4 expression in GC tissues (Figure 4F). We further assessed the mRNA and protein expression levels of KLF4 after LINC00673 was silenced in MKN-45 and SGC-7901 cells. The qRT-PCR and western blot results confirmed that the mRNA and protein expression levels of KLF4 were significantly upregulated after knockdown of LINC00673 in MKN-45 and SGC-7901 cells (Figure 5A-5B). Besides, the mRNA and protein expression levels of KLF4 were significantly increased after knockdown of EZH2 in MKN-45 and SGC-7901 cells (Figure 5C-5D). Knockdown of DNMT1 also upregulated the mRNA and protein expression levels of KLF4 in MKN-45 and SGC-7901 cells (Figure 5E-5F). These above results suggested that LINC00673 could repress KLF4 expression via interacting with EZH2 and DNMT1 in GC cells.

**Figure 6:**
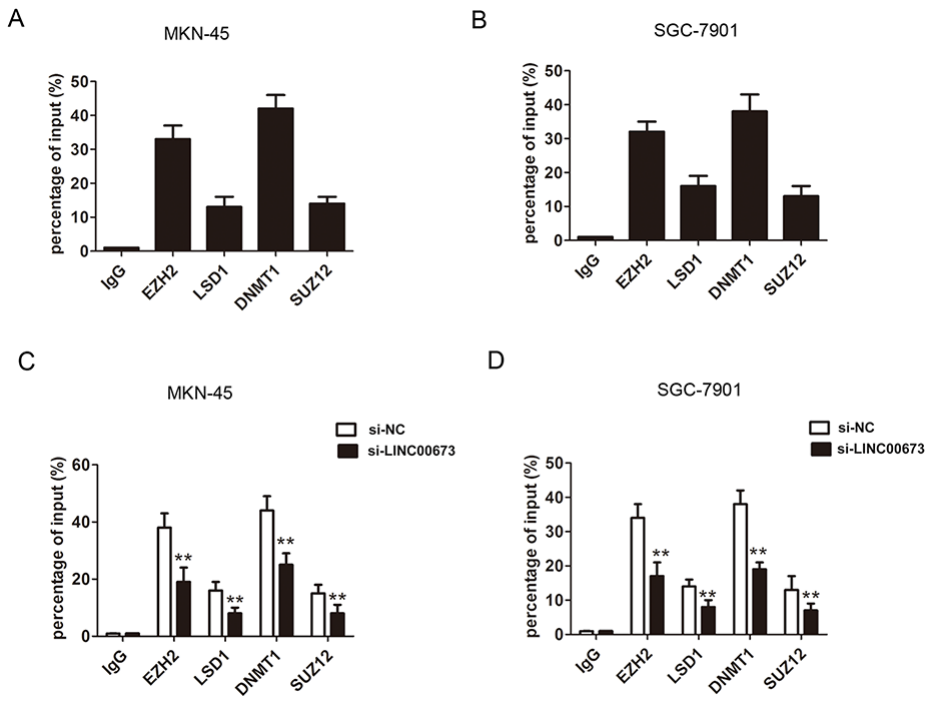
EZH2 and DNMT1 bound to promoter region of KLF4 in GC cells **(A-B)** ChIP-qPCR analysis of IgG, EZH2, LSD1, and DNMT1 and SUZ12 occupancy in the KLF4 promoter region in MKN-45 and SGC-7901 cells. **(C-D)** ChIP-qPCR analysis of IgG, EZH2, LSD1, and DNMT1 and SUZ12 occupancy in the KLF4 promoter region after LINC00673 silencing in MKN-45 and SGC-7901 cells. The mean values and SD were evaluated from triplicates of a representative experiment, ^**^p < 0.05.

To examine whether EZH2 could directly bind to the promoter region of KLF4, we constructed the primer across promoter region of KLF4. By ChIP qRT-PCR analysis, the results confirmed that EZH2 and DNMT1 could bind to the promoter region of KLF4 in MKN-45 and SGC-7901 cells (Figure 6A-6B). Meanwhile, knockdown of LINC00673 significantly reduced the binding capacity to promoter regions of KLF4 in MKN-45 and SGC-7901 cells (Figure 6C-6D). These results indicated that LINC00673 repressed KLF4 expression via directly interacting with EZH2 and DNMT1 in GC cells.

### KLF4 is partly mediated the oncogenic efficiency of LINC00673 in GC cells

**Figure 7:**
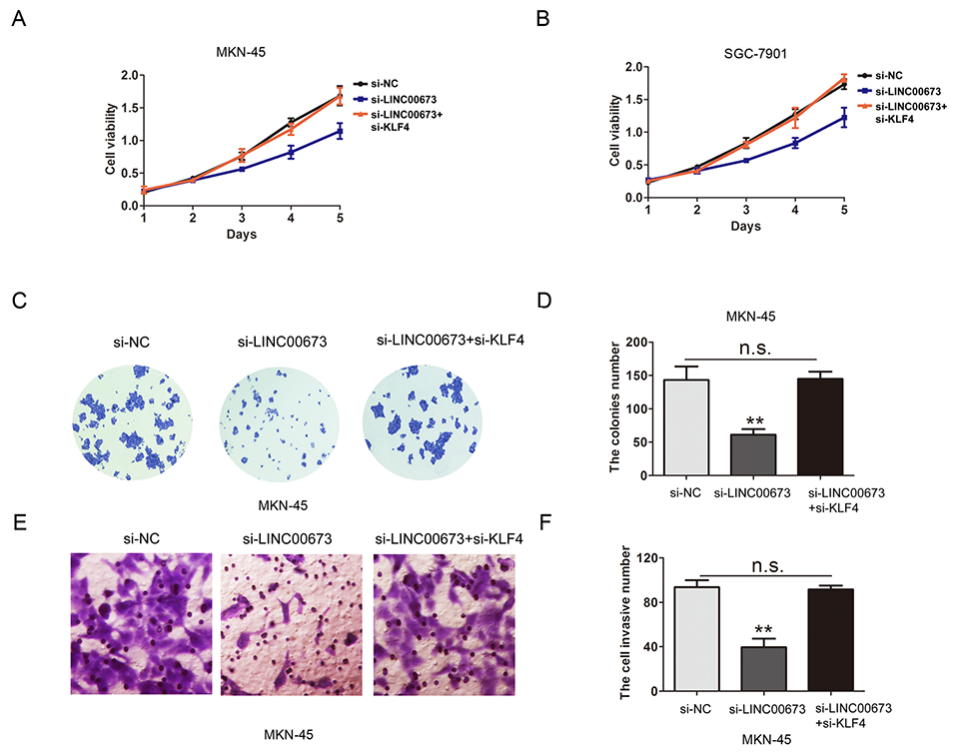
KLF4 is partly mediated in the oncogenic efficiency of LINC00673 in GC cells **(A-B)** CCK8 assay was performed to assess cell viability after transfection with si-NC, si-LINC00673 or si-LINC00673 and si-KLF4 in MKN-45 and SGC-7901 cells. **(C-D)** Cell colony formation assay was performed to assess cell proliferation after transfection with si-NC, si-LINC00673 or si- LINC00673 and si-KLF4 in MKN-45 cells. **(E-F)** Cell invasion assay was performed to assess cell invasion ability after transfection with si-NC, si-LINC00673 or si-LINC00673 and si-KLF4 in MKN-45 cells. The mean values and SD were evaluated from triplicates of a representative experiment, ^**^p < 0.05.

We evaluated whether KLF4 mediated the LINC00673 induced promoting cell proliferation and invasion in GC. By performing CCK8 assay, the results demonstrated that knockdown of LINC00673 dramatically inhibited cell viability ability in MKN-45 and SGC-7901 cells, whereas, co-transfection of si-LINC00673 and si-KLF4 reversed the suppressed effect of si-LINC00673 on cell viability (Figure 7A-7B). Furthermore, downregulation of LINC00673 significantly reduced cell colony formation number, but was rescued by co-transfection of si-LINC00673 and si-KLF4 in MKN-45 cells (Figure 7C-7D). The transwell cell invasion assay confirmed that downregulated LINC00673 decreased cell invasive number, but co-transfection of si-LINC00673 and si-KLF4 reversed the effects of si-LINC00673 on cell invasion capacity in MKN-45 cells (Figure 7E-7F). These findings indicated that KLF4 was partly mediated in the oncogenic efficiency of LINC00673 in GC cells.

## DISCUSSION

Gastric cancer represents a threat to human public health with high incidence and mortality. Recent studies have focused on that long non-coding RNAs (lncRNAs) exert essential roles in the field of tumor development and progression. LncRNAs provide some value for clinical purposes as novel biomarkers and therapeutic targets for GC patients. Higher expression of long non-coding RNA ZFAS1 is correlated with epithelial-mesenchymal transition (EMT) property of gastric cancer [15]. Long non-coding RNA linc00261 suppresses gastric cancer progression by promoting Slug degradation [16]. Helicobacter pylori infection related long non-coding RNA (lncRNA) AF147447 inhibits cell proliferation and invasion by targeting MUC2 and up-regulating miR-34c in gastric cancer [17]. Linc00152 could directly bind with EGFR which causes an activation of PI3K/AKT signaling pathway in GC [18]. In the study, we revealed that LINC00673 was dramatically higher in gastric cancer tissues compared to the normal tissues and had a positive association with lymph node metastasis, distant metastasis and advanced TNM stage in patients. Multivariate Cox model demonstrated that higher LINC00673 expression was identified as independent risk factor for prognosis in patients. These results implied that LINC00673 over-expression may be novel biological marker for predicting prognostic for GC patients.

As we known, lncRNAs was reported to be involved in the regulation of cancer cell phenotype through inhibiting the expression levels of oncogenes or tumor suppressors by a series of mechanisms, such as, chromatin remodeling, interacting with histone modification enzymes, and mediating epigenetic alteration, and so on [19, 20]. Higher PVT1 expression indicates a poor prognosis of gastric cancer patients and contributes to cell proliferation through epigenetically regulating p15 and p16 [21]. HOTAIR epigenetically silences miR34a by binding with PRC2 to promote the epithelial-to-mesenchymal transition (EMT) in human gastric cancer [22]. Long non-coding RNA HOXA-AS2 promotes gastric cancer proliferation by epigenetically silencing P21/PLK3/DDIT3 expression by binding with EZH2 (enhaner of zeste homolog 2) [11]. Long non-coding RNA HOXA-AS2 represses P21 and KLF2 expression transcription by binding with EZH2, LSD1 in colorectal cancer [23]. In the study, we demonstrated knockdown of LINC00673 suppressed cell proliferation, cell colony formation, cell migration and cell invasion in GC. RNA immunoprecipitation (RIP) and ChIP assays results found that LINC00673 promoted cell proliferation and invasion by epigenetically silencing KLF4 via binding with EZH2 and DNMT1 in GC.

KLF4 has been found to act as a tumor suppressor in GC progression, such as, MiR-32 promotes gastric carcinoma tumorigenesis by targeting Kruppel-like factor 4 and inhibits KLF4 expression in GC cells [13]. The lncRNA SNHG5/miR-32 axis promotes gastric cancer cell proliferation and migration by targeting KLF4 [24]. In the study, we demonstrated knockdown of KLF4 could reversed the effects of down-regulating LINC00673 on cell proliferation and invasion in GC cells, which indicated KLF4 was partly mediated in the oncogenic efficiency of LINC00673 in GC.

In conclusion, our results confirmed that LINC00673 expression was higher in GC and positively associated with poor survival outcome in patients. Mechanically, we confirmed that LINC00673 promoted cell proliferation and invasion by interacting with EZH2 and DNMT1 and inhibiting the KLF4 expression. Thus, inhibition of LINC00673 may be a target of treatment in GC.

## MATERIALS AND METHODS

### Patients and tissue samples

We obtained paired GC tissues and adjacent normal tissues samples from 79 GC patients who underwent primary surgical resection at affiliated Cancer Hospital & Institute of Guangzhou Medical University between January 2010 and March 2014. The GC tissues were identified as gastric adenocarcinoma and clinical data of GC patients was shown in Table 1. All of GC tissue samples were immediately frozen in liquid nitrogen and stored at -80°C until further RNA detection. No patient had received treatment before surgery. Our study protocol was approved by the Ethical and Scientific Committees of Affiliated Cancer Hospital & Institute of Guangzhou Medical University and informed consent form was obtained from all of patients.

### Cell lines culture and cells transfection

The human GC cell lines (MKN45, AGS, MKN-28 and SGC-7901) and an immortalized normal gastric epithelial cell line (GES-1) were purchased from the Institute of Biochemistry and Cell Biology of the Chinese Academy of Sciences (Shanghai, China). Cells were cultured using RPMI Medium 1640 basic media (GIBCO) and supplemented with 10% fetal bovine serum (FBS) and 100 U/ml penicillin and 100 mg/ml streptomycin (Invitrogen, Carlsbad) in a humidified incubator at 37°C with 5% CO_2_. GC cells were transfected in 6-well plates using Lipofectamine 2000 (Invitrogen, Shanghai, China), according to the manufacturers’ instructions. The siRNAs targeting LINC00673, si-negative control (NC), si-EZH2 or si-KLF4 oligos were synthesized and purchased by Shanghai GenePharma.

### Cell proliferation and cell colony formation assays

Cell viability was determined by CCK-8 cell proliferation kit (Dojin Laboratories, Kumamoto, Japan) according to the manufacturer's instructions. 2× 10^3^ cells/well were seeded in 96-well plates and cultured for 24 hours at 37°C. Cell viability was measured at 1, 2, 3, 4 and 5 days, respectively. CCK-8 reagent was added to each well contained 100 μL of the culture medium, and then incubated for 2 h at 37°C. Cell proliferation was evaluated and the absorbance at 450 nm by a microplate reader. For cell colony formation assay, transfected cells (300 cells/well) with si-NC, si-LINC00673 or si-KLF4 were plated in 12 well plates and cultured in RMPI-1640 medium for 2 weeks. Cells colonies were stained with crystal violet and were counted and photographed for statistical analysis. All procedures were carried out in triplicate.

### Cell invasion assay

For cell invasion assays, transwell membranes were precoated with Matrigel (BD bioscience, Franklin Lakes, USA) and cells were incubated for 24 hour at 37 °C in a 5% CO_2_ atmosphere. 1.5 × 10^5^ cells transfected with si-NC, si-LINC00673 or si-KLF4 in 400 μL serum-free medium were plated on the top chamber and 500 μL DMEM medium with 10% high concentration of serum was added on the bottom chamber. The GC cells on the upper chamber were carefully removed and cells on the lower chamber were stained for 30 min with 0.1% crystal violet. Cells were counted in ten random fields. The experiment was independently repeated at least three times.

### RNA extraction and quantitative real-time PCR (qRT-PCR) analysis

The total RNA sample collected from GC tissue and adjacent normal tissues samples or GC cells was extracted using the Trizol reagent (Invitrogen, Carlsbad, CA, USA) according to the manufacturer's protocol. Reverse transcription of 1 μg RNA was performed using Moloney murine leukaemia virus (MMLV) reverse transcriptase kit (Takara, Japan) according to the manufacturer's protocol. Quantitative real-time PCR was carried out on the ABI 7500 quantitative PCR System (Applied Biosystems, Foster City, CA, USA) according to the manufacturer's instructions. The mRNA expression was calculated using 2^−ΔΔCt^ methods. Experiments were repeated at least three times.

### Western blot analysis

The proteins were extracted as previously described [25]. Primary antibodies including PCNA antibody (1:500, Santa Cruz Biotechnology, CA, USA), CyclinD1 antibody (1:500, Santa Cruz Biotechnology, CA, USA), CDK2 (1:1000, Santa Cruz Biotechnology, CA, USA), KLF4 (1:1000, Santa Cruz, CA, USA) and GAPDH antibody (1:3000, Abcam, USA) were used in the study. The western blot bands were evaluated by Image J software (National Institute of Mental Health, Bethesda, Maryland, USA).

### RNA binding protein immunoprecipitation (RIP) assay

RNA immunoprecipitation (RIP) experiment was performed by using a Magna RIP RNA-Binding Protein Immunoprecipitation Kit (Millipore, USA) following the manufacturer's instructions. MKN-45 and SGC-7901 cells were lysed and incubated with protein A Sepharose beads which were conjugated using antibodies with IgG, EZH2, DNMT1, LSD1 and SUZ12 (Santa Cruz, USA) at 4°C. The immunoprecipitated RNA was purified and detected by qRT-PCR.

### Chromatin immunoprecipitation(ChIP) assay

ChIP experiments were performed as described previously [26]. Briefly, cells were cross-linked with 1% formaldehyde for 10 min at room temperature and cross-linked cells were lysed using lysis buffer and sonicated for 30 min. Then, Sonicated lysates were immunoprecipitated using antibodies with EZH2, DNMT1, LSD1, SUZ12 (Santa Cruz, USA) and IgG (Cell Signaling Technology, USA) antibodies.

### Statistical analyses

All experiments were independently performed at least three times and statistical analysis was evaluated by SPSS version 20.0 software (SPSS Inc. Chicago, IL, USA). Differences between two independent groups were analyzed by Student's t-test or ANOVA. The results were showed as mean ± SD. P-values of less than 0.05 were considered to be statistically significant.
